# Quality of life assessment in diabetic patients: validity of the creole version of the EQ-5D-5L in Reunion Island

**DOI:** 10.3389/fpsyg.2023.1185316

**Published:** 2023-06-15

**Authors:** Maissa Safieddine, Lea Bruneau, Ibtissame Soulaimana, Xavier Debussche, Sophie Lafarge, Bruno Falissard, Cyril Ferdynus, Laetitia Huiart

**Affiliations:** ^1^Centre Hospitalier Universitaire de La Réunion, Saint-Denis, France; ^2^Inserm CIC 1410, Centre Hospitalier Universitaire de La Reunion, Saint Pierre, Réunion; ^3^French Public Health, Mamoudzou, Mayotte; ^4^Department of Endocrinology, Centre Hospitalier Universitaire de la Reunion, Saint Denis, Réunion; ^5^Epidemiological and Public Health Research Centre, Villejuif, France; ^6^INSERM U1153, Centre de Recherche Épidémiologie et Statistique, Paris, France; ^7^French Public Health, Saint Maurice, France

**Keywords:** HRQOL, EQ-5D-5L, creole-translation, type II diabetes, Reunion Island

## Abstract

**Introduction:**

Due to the high prevalence of diabetes and its complications, evaluating the patient’s quality of life is critical. EQ-5D-5L is a valid tool for assessing health-related quality of life (HRQOL) in chronic diseases, including diabetes. However, no psychometric measures have been validated in a Creole-speaking population. Therefore, this study aimed for the first time to validate and cross-culturally adapt Creole and French versions of EQ-5D-5L on Type II diabetes patients in Reunion Island.

**Materials and methods:**

The Creole translation and cross-cultural adaptation process were based on the EUROQOL methods. Internal consistency and construct validity were determined using confirmatory factor analysis (CFA) of EQ-5D-5L for both versions. CFA model for HRQOL and global fit measures were calculated based on the EQ-5D-5L items using the maximum likelihood (ML) method.

**Results:**

From November 2016 to October 2017, 148 patients were included in the Creole group and 152 in the French group. EQ-5D-5L measures were unidimensional for both versions. Cronbach’s coefficient alpha was 0.76 for the Creole version and 0.81 for the French version in CFA models. The root mean square error of approximation (RMSEA) was 0.06 for the Creole version and 0.02 for the French version. The Comparative Fit Index (CFI) was closer to 1 for both versions. CFA models for both the Creole and French versions fit the data adequately.

**Discussion:**

Overall, our findings provided evidence that both the Creole and French versions of EQ-5D-5L are suitable for assessing HRQOL in diabetes patients in Reunion Island. However, further research could be done to investigate French–Creole differences concerning the perception of health status, and a cultural adaptation of the French version will be considered.

## Introduction

Quality of life is increasingly being used as an outcome for the assessment of health care interventions (Roper et al., 1988). There is a growing interest in tools that can be used to measure health-related quality of life (HRQOL) in general population surveys, as well as across a variety of diseases. Two types of tools have been developed to measure HRQOL. Generic tools are general purpose measures used to assess HRQOL of communities and for comparison between populations. Disease-specific tools focus on particular diseases and can be useful for assessing treatment effectiveness. Among the generic tools, the EQ-5D provides a simple generic measure of health for clinical and economic appraisal. This generic scale is a two-part, preference-based measure of health-related quality of life developed by the EuroQol Research Foundation (2022).

International interest in this questionnaire led to its translation into more than 170 languages and is widely used in many different countries in a variety of clinical areas and evaluation programs, as well as in health policy formulation (Poór et al., 2017; EuroQol Research Foundation, 2022). The instrument has been translated into French as well and has been adapted to the stated preferences of the French population facilitating cost-effectiveness analysis (Rabin and de Charro, 2001; Andrade et al., 2020). Reunion Island is a French island located in the Indian Ocean. Its population is characterized by cultural and religious diversity, and both the Creole and French languages are spoken. Creole is mainly derived from French but includes terms from many other languages (Malagasy, Hindi, Portuguese, Gujarati, and Tamil) (Merlo, 1975). Creole is primarily a spoken language and is rarely written.

However, one could ask if the French version of EQ-5D-5L is suitable for the specific population of Reunion Island that is mainly creole-speaking.

Chronic diseases like diabetes mellitus are known to compromise the HRQOL. Type 2 Diabetes mellitus (DM) is a chronic metabolic disease known to affect HRQOL adversely (Diabetes Care, 1999; Wee et al., 2005; Wexler et al., 2006; Chevalier and de Pouvourville, 2013). The prevalence of diabetes in Reunion Island is estimated to be 10%, almost twice as much as in mainland France (10% versus 5% in 2015) (Coffey et al., 2002). Since this chronic disease impact quality of life (Fagot-Campagna et al., 2010), accurate evaluation is needed in this population of patients. However, the lack of psychometric tools specifically validated for Reunion Island’s population has been a major difficulty in conducting clinical and epidemiological research focused on quality of life.

Thus, the first aim of this study aim was to translate and adapt a Creole version of EQ-5D-L and to evaluate its psychometric properties on patients with type II diabetes in Reunion Island. And the secondary aims were to assess and compare the psychometric properties of the EQ-5D-5L (in Creole and French) in two distinct populations.

## Methods

### Instrument

The EQ-5D consists of two parts. The first part is the EQ-5D-5Ldesscriptive system, composed of five dimensions; each dimension has one item/question. The five items are mobility, self-care, usual activities, pain/discomfort, and anxiety/depression. Each item can be answered on a 5-point scale: no problems, slight problems, moderate problems, severe problems, and extreme problems. A dimension in which the patient responded “no problem” is said to be at level 1, while a dimension in which the patient responded “extreme problem” is said to be at level 5. The total score for the EQ-5D-5L questionnaire is calculated by combining the scores for each of the five dimensions. The total score can range from 5 (no problems in any dimension) to 25 (extreme problems in all dimensions).

The second part is the EQ-5D visual analog scale (VAS), which records the patient’s self-rated health on a vertical axis from 0 to 100. Zero represents the worst possible health state, while 100 represents the best possible health state. The subject marks a point on the scale to assess the global level of health.

### Translation and cultural adaptation methods

**Figure 1:**
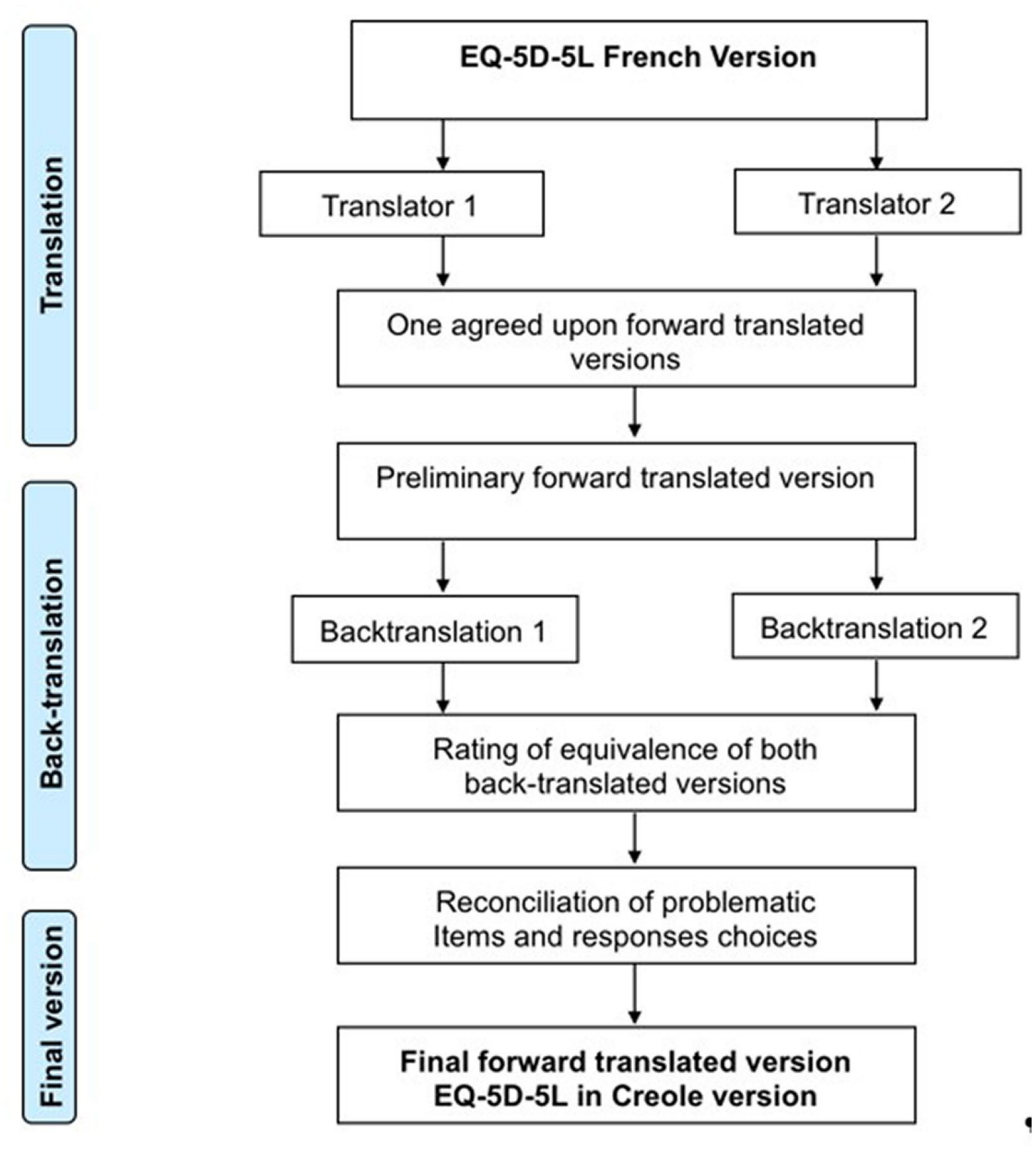
Translation procedure of the Creole EQ-5D-5L.

Translation and cross-cultural adaptation processes were in accordance with the EuroQol Research Foundation (2022). Because Creole is mainly derived from French, we decided to use the French version of the EQ-5D-5L questionnaire as the template from which we would translate and adapt into Creole. After authorization from EuroQol group, the French EQ-5D-5L was adapted and translated into Creole (Figure 1).

The original French language questionnaire was translated by two independent translators who are native speakers of the target language (Creole) and fluent in French. This translation was associated with a cultural adaptation of questions and answers. Translators rated the difficulty of translating each item and response choice using a rating scale ranging from 0 (not at all difficult) to 100 (extremely difficult) and provided comments as appropriate (IDF diabetes atlas, 2017).

The extent of agreement between raters was evaluated by calculating the intra-class correlation coefficients (ICC) and their 95% confidence intervals (CI). An ICC >0.5 was considered acceptable and an ICC > 0.7 was considered as good (Bullinger et al., 1998).

After review by an expert committee composed of two linguistic experts, two translators and one expert researcher in psychometric scales, the preliminary version was edited and sends to EuroQol group to obtain their agreement to proceed to the next step. This first version was then translated back to French by two independent bilingual translators who were native French speakers and fluent in Creole. These translators rated the quality of the agreement of the forward translation on a scale from 0 (not at all perfect) to 100 (perfect). Backward-translations of EQ-5D-5L Creole were reviewed by the expert committee. The pre-final version was edited and tested on a population of 30 patients. Each subject had to answer the questionnaire and asked about difficulties encountered in understanding the items. This allowed us to identify inconsistencies, which were not raised with the experts.

The final version of the EQ-5D-5L in Creole was then obtained thanks to the information obtained through the previous steps and was then approved by the expert committee.

### Participants and procedures

The study was conducted from November 2016 to October 2017. The participants’ eligibility criteria for the French and Creole version were identical. Participants were eligible for inclusion if they were at least 18 years old, were diagnosed with type II diabetes for at least 1 year, and lived in Reunion Island. In addition, for the Creole version, participants should speak Creole and have resided in Reunion for at least 5 years. We excluded participants who had cognitive disturbances, could not understand and respect the study procedures, or had pre-existing medical conditions that limited objective assessment after surgery and the antecedent of a cerebrovascular accident with neurological or motor disability. Written informed consent was obtained from all enrolled subjects.

Patients were recruited during consultations to monitor of their diabetes in two diabetology departments of the University Hospital of Reunion Island (Saint-Denis and Saint-Pierre). Patients self-completed the questionnaire or were helped by a Creole-speaker technician for the Creole version to not exclude illiterate populations. Participants also filled in a small socio-demographic and clinical questionnaire.

### Statistical analyses

We estimated descriptive statistics to compare the socio-demographic and clinical characteristics of patients in the Creole and French version. Patient characteristics are presented as mean and standard deviation (SD) in the case of continuous data, and absolute and relative frequencies in the case of categorical data. Pearson’s chi-squared and T-student tests were performed to determine statistical significance. For the Creole and French versions, we calculated the mean of EQ-5D-5L VAS and the SD.

Validity assessment of the EQ-5D-5L in Creole and French has been evaluated by means of content, construct, and criterion validity (Fermanian, 1984). Floor and ceiling effects for all items were assessed using bar graphs. Analyses of psychometric properties were realized independently for Creole and French version.

### Internal consistency

Internal consistency was measured for the EQ-5D-5L using Cronbach’s coefficient alpha, as a typically reported measure for associations between multi-item scales. Cronbach’s alpha coefficient of 0.70 or higher was considered satisfactory (Fermanian, 1984; Gandek and Ware, 1998).

### Construct validity

A confirmatory factor analysis (CFA) model was carried out to examine the construct validity of the EQ-5D-5L items for mapping HRQOL in diabetes patients (Gandek and Ware, 1998). The number of factors was determined based on the screen plot, originally proposed by Cattell (1966) and Schermelleh-Engel et al. (2003). To estimate the parameters of the CFA model, the maximum likelihood (ML) method was applied (Cattell, 1966; Schermelleh-Engel et al., 2003). Local fit measures (explained variance; average variance; correlation coefficient; standardized regression coefficient; factor reliability) (Olsson et al., 2000; Kline, 2005) and global fit measures (chi-squared statistic; standardized root mean square residual, SRMR; root mean square error of approximation, RMSEA; Comparative Fit Index, CFI; Tucker-Lewis Index, TLI) (Cattell, 1966; Olsson et al., 2000; Sousa and Kwok, 2006) were calculated. Model fit was checked if the SRMR was less than 0.8 (Hooper et al., 2007) and CFI is greater than 0.95 (Hu and Bentler, 1999). In addition, the model fit was considered good if the RMSEA was equal or less than 0.05 and excellent with an RMSEA equal to 0.01 (MacCallum et al., 1996).

The CFA models were performed using R statistical software version 3.5.3 (packages: prettyR, psy,dplyr, Amelia, Hmisc, lavaan, cocron, tableone) (Bentler, 1990; Arifin, 2018).

## Results

### Translation and cultural adaptation of the EQ-5D into creole

Strictly following the previously detailed procedure, the EQ-5D-5L was translated into Creole language without any major difficulties. The Median difficulty score between two raters with response choices was 3 for Q1-Mobility, 3 for Q2-Self-Care, 35 for Q3-Usual Activities, 47 for Q4-Pain/Discomfort and 65 for Q5-Anxiety/Depression. The ICC between two raters was 0.41 [−0.05, 0.72] in response choices difficulty. Median items clarity ratings was 40.0 (IQR: 37.5–45.0) and median responses clarity ratings was 90.0 (IQR: 70–100). In response choices clarity, the ICC between two raters was 0.07 [−0.35, 0.45].

### Clinical characteristics in creole and French versions

Validation of EQ-5D-5L in the Creole and French languages was conducted using two independent samples including 148 and 152 subjects, respectively, in each group. Two subjects in Creole version did not meet the inclusion criteria and were excluded from the data analysis. The demographics and clinical characteristics among groups are summarized in Table 1. The mean age was 57.1 years for Creole version and 56.5 years for the French version. In both groups, the majority of patients were females (52.1% versus 57.9%) and they had a duration of diabetes type 2 equal to 5 years and above (83.1% versus 85.5%). The mean EQ-5D-5L VAS score for French version was 67.3 ± 19.5 and 64.4 ± 22.8 for Creole version.

**Table 1:** Socio-demographic and clinical characteristics of patients.

Socio-demographic variables	EQ-5D Creole version (*N* = 148)	EQ-5D French version (*N* = 152)	Value of *p*
Mean age (SD), years	57.1 (10.5)	56.5 (11.9)	0.654
Gender [*n* (%)]			
Female	77 (52.1%)	88 (57.9%)	
Male	71 (47.9%)	64 (42.1%)	0.365
Professional Status [*n* (%)]			0.618
Employed	45 (30.4%)	39 (25.7%)	
Retired	41 (27.7%)	46 (30.3%)	
Unemployed	62 (41.9%)	66 (43.4%)	
Missing	0	1 (0.7%)	
Education level [*n* (%)]			0.488
Out-of-school	5 (3.4%)	8 (5.3%)	
Elementary school	52 (35.1%)	43 (28.3%)	
Middle school	42 (28.4%)	42 (27.6%)	
High school	31 (20.9%)	29 (19.1%)	
College and above	16 (10.8%)	24 (15.8%)	
Other	2 (1.4%)	5 (3.3%)	
Missing	0	1 (0.7%)	
Duration of diabetes			0.518
Between 1 and 2 years	10 (6.8%)	6 (3.9%)	
Between 2 and 5 years	14 (9.5%)	16 (10.5%)	
5 years and above	123 (83.1%)	130 (85.5%)	
Missing	1 (0.7%)	0	
BMI (kg/m^2^)			0.554
Underweight	0 (0.0%)	2 (1.3%)	
Normal	24 (16.7%)	25 (16.7%)	
Overweight	49 (34.0%)	60 (40.0%)	
Obese I	40 (27.8%)	32 (21.3%)	
Obese II	20 (13.9%)	19 (12.7%)	
Obese III	11 (7.6%)	12 (8.0%)	
Diabetes complications			
Severe foot injury	10 (6.8%)	8 (5.3%)	0.127
Retinopathy	17 (11.5%)	18 (11.8%)	0.171
Comorbidities			
Acute coronary accident	8 (5.4%)	25 (16.4%)	0.005
Ischemic Stroke	5 (3.4%)	13 (8.6%)	0.129
Angor	3 (2.0%)	6 (3.9%)	0.166
Coronary disease	10 (6.8%)	11 (7.2%)	0.228
Heart failure	1 (1.0%)	6 (3.9%)	0.052
High blood pressure	80 (54.1%)	101 (66.4%)	0.089
Neuropathy of lower limb	24 (16.2%)	35 (23.0%)	0.110
Arteriopathy	9 (6.1%)	11 (7.2%)	0.178
Macro-proteinuria	15 (10.1%)	24 (15.8%)	0.102
End-stage renal failure	35 (23.6%)	41 (27.0%)	0.179
Other nephropathy	15 (10.1%)	9 (5.9%)	0.043
Management of diabetes			
Dietary rules	139 (93.9%)	149 (98.0%)	0.128
Oral antidiabetic drugs	126 (85.1%)	123 (80.9%)	0.413
Non-insulin injections	41 (27.7%)	39 (25.7%)	0.787
Insulin	74 (50.0%)	95 (62.5%)	0.049

### Analyses of item quality

**Figure 2:**
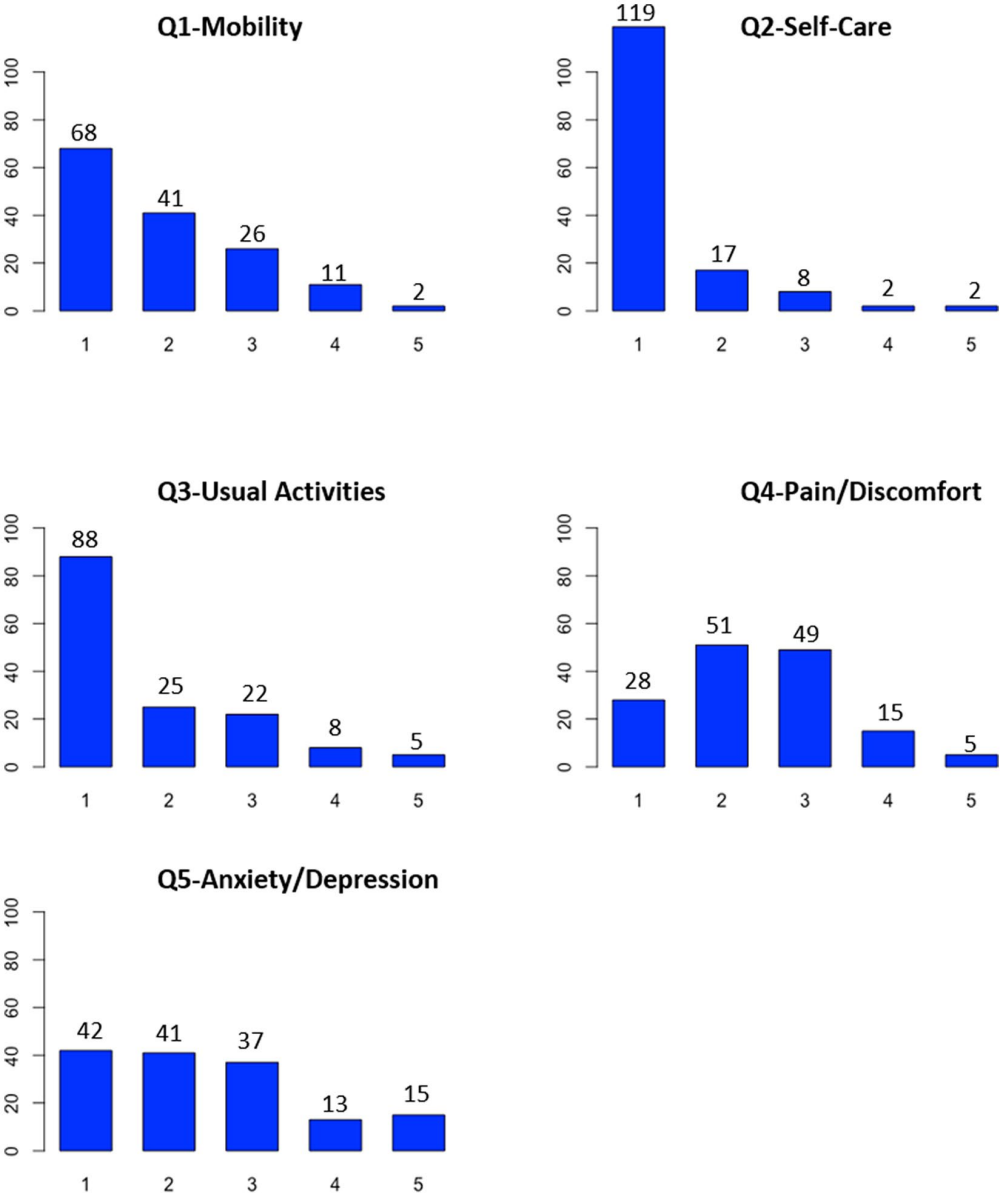
Quality of items of the Creole version of EQ-5D-5L.

**Figure 3:**
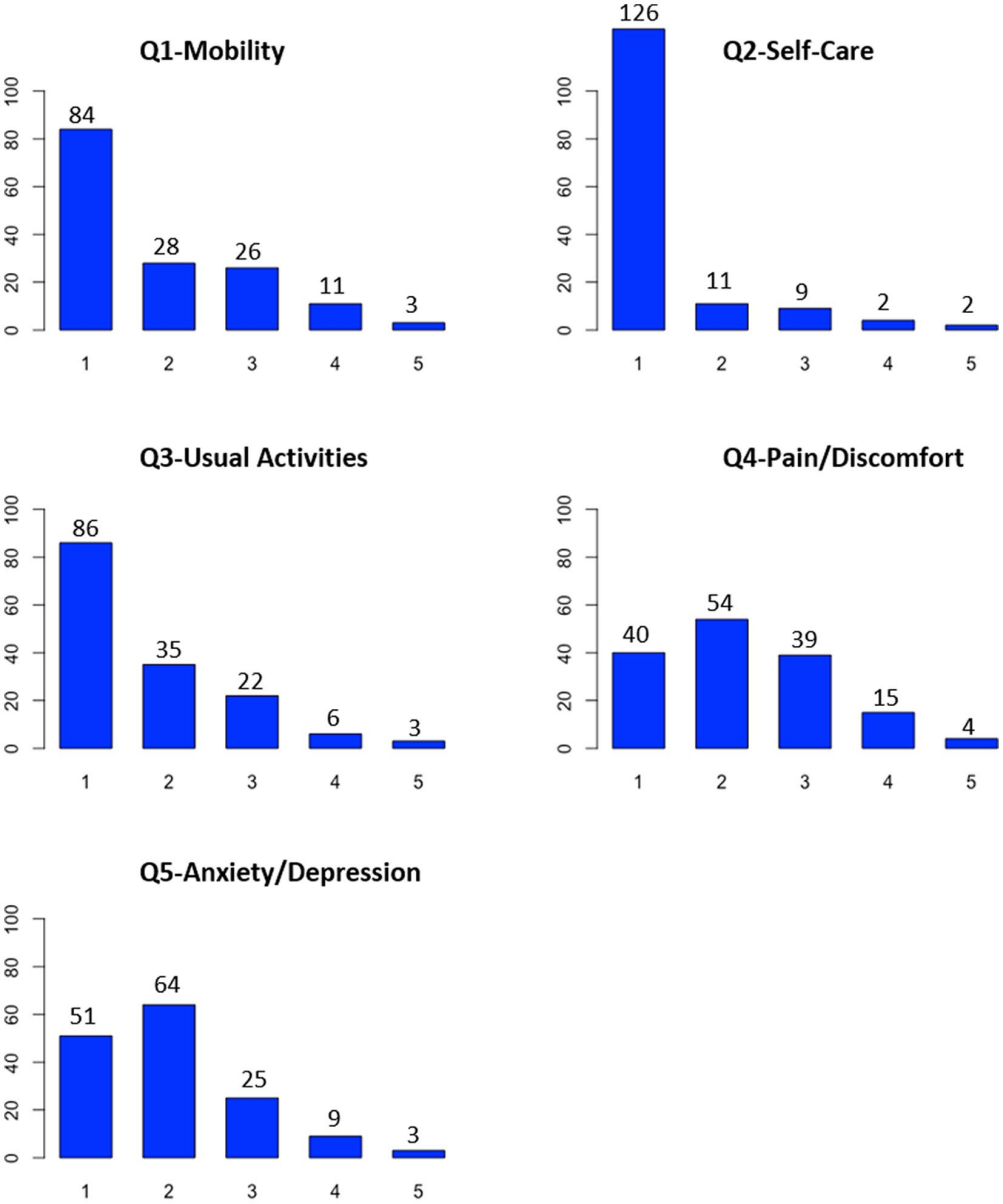
Quality of items of the French version of EQ-5D-5L.

At baseline, a floor effect was observed for Q2-Self-Care in Creole and French versions, and no ceiling effects were observed in each item for both versions (Figures 2, 3). There was no missing data at baseline. Therefore, the quality of the items in both versions appeared to be correct at baseline.

### Internal consistency reliability

Cronbach’s coefficient alpha was 0.76 for Creole version and 0.81 for French version. Then internal consistency reliability of the EQ-5D-5L was good in both versions with Cronbach’s alpha coefficients exceeding the recommended 0.70 standard for group-level comparisons.

### Construct validity

The scree plots are presented in Supplementary Figures S1, S2 for the Creole and French versions. With parallel analysis method, both figures showed that only one dimension emerges clearly compared to the others. Therefore, at baseline, EQ-5D-5L scale was unidimensional in the French and Creole versions.

**Figure 4:**
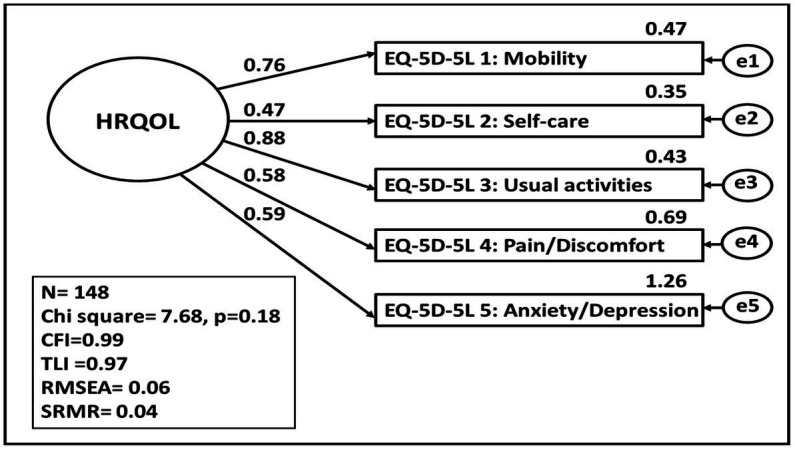
HRQOL represented by the EQ-5D-5L items in Creole version. Basic measurement model of the latent construct of HRQOL with model fit and standardised parameter estimates calculated using the maximum likelihood method. CFI, Comparative Fit Index; TLI, Tucker Lewis Index, REMSEA, root mean square error of approximation, SRMR, root mean square error of approximation.

**Figure 5:**
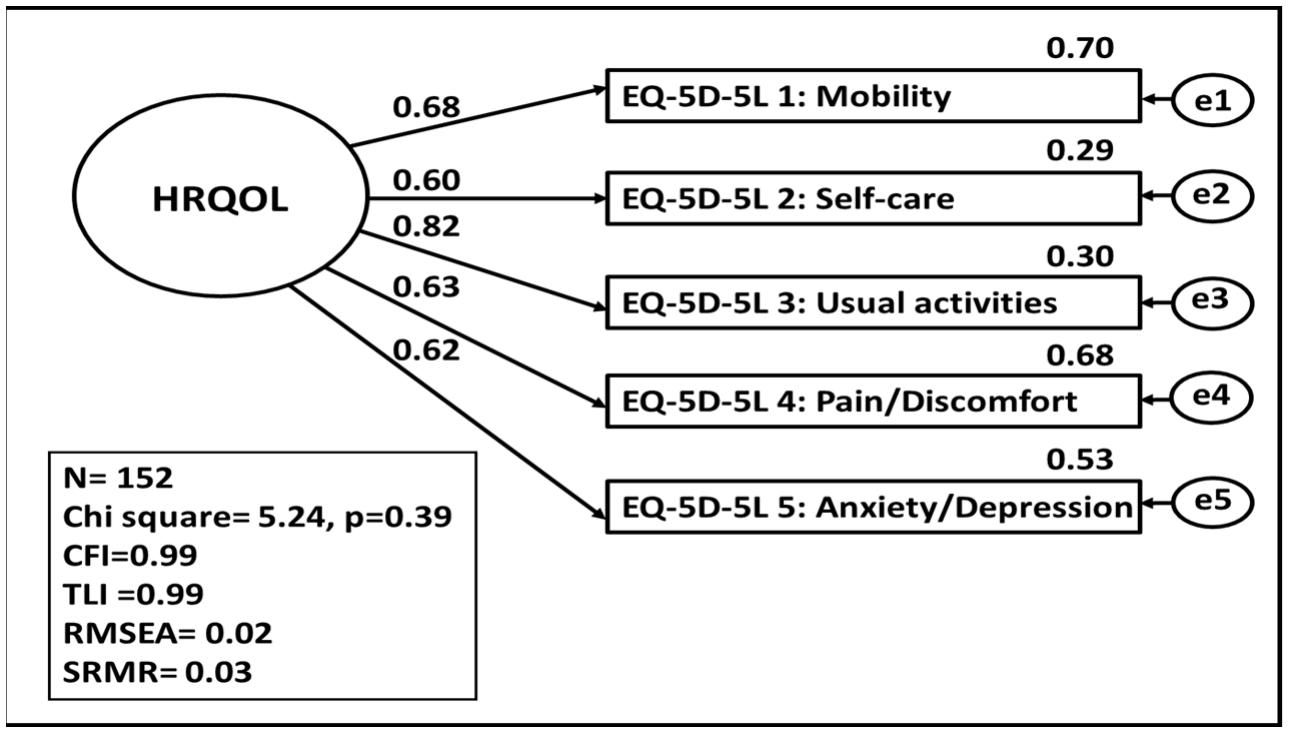
HRQOL represented by the EQ-5D-5_5L items in French version. Basic measurement model of the latent construct of HRQOL with model fit and standardised parameter estimates calculated using the maximum likelihood method. CFI, Comparative fit index; TLI, Tucker Lewis Index, REMSEA, root mean square error of approximation, SRMR, root mean square error of approximation.

The CFA models are presented in Figures 4, 5 for the Creole and French versions. The standardized regression coefficients of the EQ-5D-5L items for the latent construct HRQOL as well as the explained variances are shown in these two figures.

For the Creole version, the factor loadings for “usual activities,” “mobility,” “anxiety/depression,” and “pain/discomfort” are sufficient, whereas the factor loading for “self-care” is low. The RMSEA is equal to 0.06, indicating a good approximate fit. CFI is closer to 1 and SRMR is equal to 0.04, indicating a good fit. All the explained variance of the items revealed an acceptable values threshold. Thus, the CFA model fits the data adequately according to the model fit statistics.

For the French version, the factor loadings for all items on the EQ-5D-5L are sufficient. All the explained variance of the items revealed an acceptable values threshold. According to the model fit statistics (RMSEA =0.02, CFI = 0.99, SRMR =0.03), the CFA model fits the data adequately.

## Discussion

The French and Creole versions of the EQ-5D-5L proved to be a reliable and valid method for measuring the quality of life of patients from Reunion Island with type II diabetes. The Creole version of the short EQ5D-5 L was completed by 148 patients, while the French version was completed by 152 patients. Our study found that the EQ-5D-5L is a suitable short generic instrument for assessing patients with diabetes. Furthermore, construct validity was found for all parameters of the EQ-5D-5L.

Our study aimed to translate and culturally adapt the French EQ-5D-5L into Creole following the EUROQOL method and evaluate its psychometric properties on Reunionese patients with type II diabetes. The translation and cultural-adaptation methodologies used were similar to those used for the French translation. The difficulty rating results obtained during our study showed that translators did not encounter great difficulties when translating the French EQ-5D-5L into Creole and the ICC between the two raters was acceptable in response choices difficulty. However, the clarity of translation was rated as poor and the ICC between the two raters in response choices clarity was considered poor. The results of the difficulty and of the quality ratings of the translation in this study cannot be compared with other studies since these results varied considerably over countries. Moreover, the Creole EQ-5D-5L version showed acceptable comprehensibility and face validity. Thus, this reflects the difficulty of translating from French into written Creole since Creole primarily exists as an oral language.

In the present study, a floor effect for Q2-Self-Care was found in both versions, which means that EQ5D may not be sensitive enough to detect differences in self-care status among individuals. The absence of floor and ceiling effects in the four other items for both the Creole and French versions indicated acceptable measurement standards. Moreover, the internal consistency and reliability of both the Creole and French EQ-5D-5L versions were good, as shown in other psychometric studies using EQ-5D in diabetes patients (Hazaël-Massieux, 2001). For the Creole and French versions, the EQ-5D-5L scale was unidimensional, and the CFA models fit the data adequately according to the model fit statistics.

Thus, there is mixed evidence for the internal construct validity of the EQ-5D-5L. In both versions, there was an explained variance below threshold concerning the questions on self-care and usual activities (Gebremariam et al., 2022). This finding could be related to the health status of patients with diabetes. This is shown in Scandinavian studies which found that, when compared with controls, patients with diabetes reported lower well-being (Clouth et al., 2008) and health limitations that affected their mobility and activities of daily living (Naess et al., 1995). Another possible explanation is the lifestyle of the population in Reunion Island. Indeed, the lifestyle of the population has undergone rapid westernization since the departmentalization of the island in 1946. This westernization is at the origin of an unbalanced diet rich in sugar and fat and a decrease in physical exercise (Gåfvels et al., 1991). In fact, 60% of the population of Reunion is below the world’s recommendations regarding physical exercise Tableau de bord sur le diabète à La Réunion, 2015. In the Creole version of the EQ-5D-5L, the anxiety/depression question has an explained variance greater than 1. This result reflects the translator’s feelings as they rated the translation of this item as extremely difficult. Thus, the anxiety/depression question in the Creole version should be further investigated.

### Strengths and limitations

To the best of our knowledge, this is the first time that the EQ-5D-5L has been validated in a sample of creole-speaking patients. This validation of the scale in Creole is preliminary, and other psychometric properties should be evaluated. One limitation is that our study is limited to patients with diabetes type 2. Hence, the findings cannot be generalized to all of the Reunion population. However, this study shows that the EQ-5D-5L is a reliable tool in measuring HRQOL of type 2 diabetes patients. The tool can be explored further to assess the quality of life of the Reunion population and to compare it with patients suffering from chronic diseases.

## Conclusion

The instrument is valid and reliable. Overall, our findings provided evidence that both the Creole and French versions of the very short generic EQ-5D-5L are suitable tools for assessing HRQOL in diabetic patients in Reunion Island. Globally, both versions have the same psychometric properties. However, investigators felt the French version was easier to administrate than the Creole version. Further research can be done to investigate French–Creole differences in the perception of health status and a cultural adaptation of the French version will be considered.

## Data availability statement

The raw data supporting the conclusions of this article will be made available by the authors, without undue reservation.

## Ethics statement

The studies involving human participants were reviewed and approved by the Comités de protection des personnes (CPP). The patients/participants provided their written informed consent to participate in this study.

## Author contributions

MS: formal analysis and writing—original draft. CF: supervision, validation, visualization, and review—editing. LB: visualization and review—editing. XD: resources. SL: project administration. BF: visualization and review—editing. LH: methodology, supervision, and funding acquisition. All authors provided critical revision of the manuscript for important intellectual content and approved the version to be published.

## Conflict of interest

The authors declare that the research was conducted in the absence of any commercial or financial relationships that could be construed as a potential conflict of interest.

## Publisher’s note

All claims expressed in this article are solely those of the authors and do not necessarily represent those of their affiliated organizations, or those of the publisher, the editors and the reviewers. Any product that may be evaluated in this article, or claim that may be made by its manufacturer, is not guaranteed or endorsed by the publisher.
