# Supplementary material for: Quality of life assessment in diabetic patients: validity of the creole version of the EQ-5D-5L in Reunion Island
Source: Front Psychol. 2023 Jun 15;14:1185316. doi: 10.3389/fpsyg.2023.1185316 (PMC10311213; doi:10.3389/fpsyg.2023.1185316)
Supplement: Supplementary file 1 [file Table_1.DOCX]

**SUPPLEMENTARY**

**Figure S1.** Scree plot of the Creole version of EQ-5D-5L

**
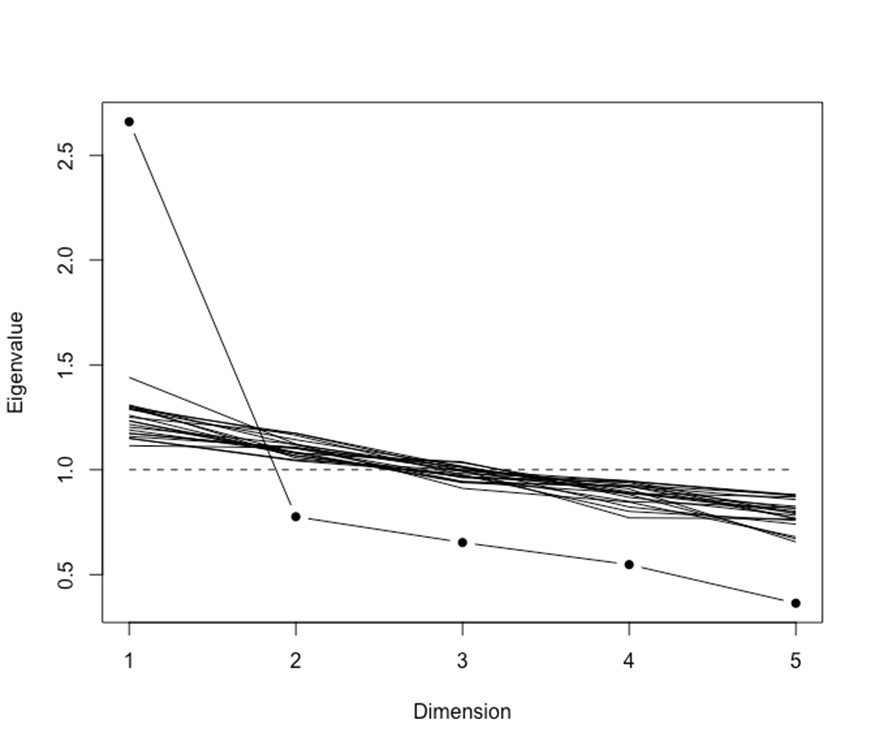
**

**Figure S2.** Scree plot of the French version of EQ-5D-5L

**
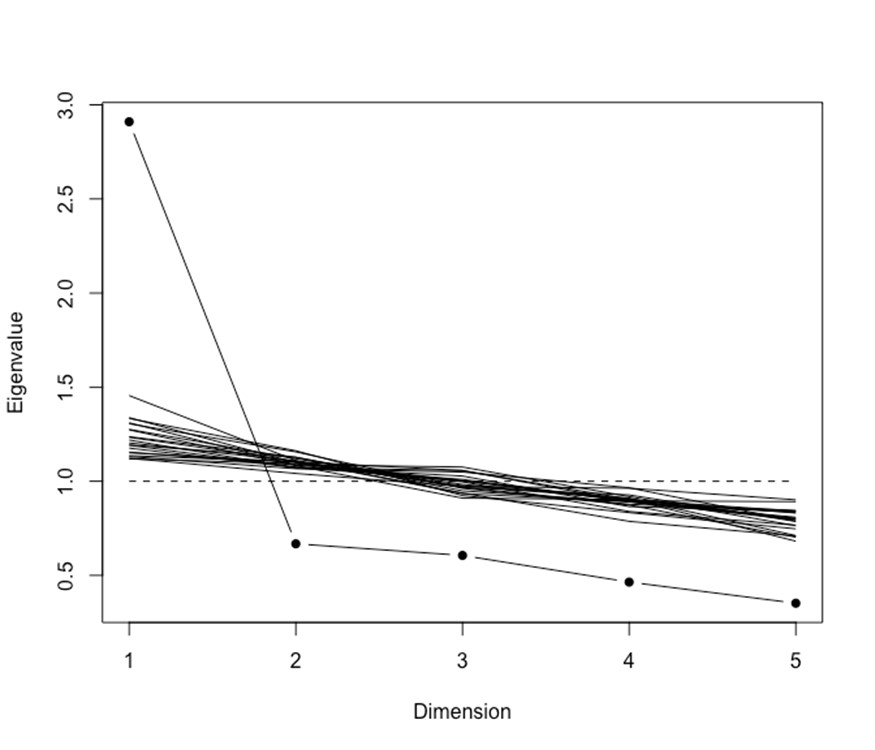
**

**Table S1.** Measures of global fit for the CFA models for Creole and French versions of EQ-5D-5L

|  | **Valid n** | **Chi^2^** | **Df** | **P-value** | **TLI** | **CFI** | **RMSEA** | **SRMR** |
| --- | --- | --- | --- | --- | --- | --- | --- | --- |
| **Thresholds for model acceptance** |  |  |  | **>0.05** | **≥ 0.90** | **> 0.95** | **≤ 0.080** | **<0.08** |
| **Maximum likelihood method** |  |  |  |  |  |  |  |  |
| **Creole version** | **148** | **7.68** | **5** | **0.175** | **0.971** | **0.986** | **0.06** | **0.036** |
| **French version** | **152** | **5.25** | **5** | **0.386** | **0.998** | **0.999** | **0.018** | **0.025** |

CFA: confirmatory factor analysis, Df: degrees of freedom, TLI: Tucker Lewis Index, CFI: Comparative Fit Index; REMSEA: root mean square error of approximation, SRMR: root mean square error of approximation
